# Simultaneous bioremediation of phenol and tellurite by *Lysinibacillus* sp. EBL303 and characterization of biosynthesized Te nanoparticles

**DOI:** 10.1038/s41598-023-28468-5

**Published:** 2023-01-23

**Authors:** Firooz Hosseini, Elham Lashani, Hamid Moghimi

**Affiliations:** Department of Microbial Biotechnology, School of Biology, College of Science, University of Tehran, Tehran, 1417864411 Iran

**Keywords:** Biotechnology, Microbiology, Environmental sciences, Natural hazards, Nanoscience and technology

## Abstract

Aromatic compounds and metalloid oxyanions are abundant in the environment due to natural resources and industrial wastes. The high toxicity of phenol and tellurite poses a significant threat to all forms of life. A halotolerant bacterium was isolated and identified as *Lysinibacillus* sp. EBL303. The remediation analysis shows that 500 mg/L phenol and 0.5 mM tellurite can be remediated entirely in separate cultures within 74 and 56 h, respectively. In addition, co-remediation of pollutants resulted in the same phenol degradation and 27% less tellurite reduction within 98 h. Since phenol and tellurite exhibited inhibitory behavior, their removal kinetics fitted well with the first-order model. In the characterization of biosynthesized tellurium nanoparticles (TeNPs), transmission electron microscopy, dynamic light scattering, FE-SEM, and dispersive X-ray (EDX) showed that the separated intracellular TeNPs were spherical and consisted of only tellurium with 22–148 nm in size. Additionally, investigations using X-ray diffraction and Fourier-transform infrared spectroscopy revealed proteins and lipids covering the surface of these amorphous TeNPs. Remarkably, this study is the first report to demonstrate the simultaneous bioremediation of phenol and tellurite and the biosynthesis of TeNPs, indicating the potential of *Lysinibacillus* sp. EBL303 in this matter, which can be applied to environmental remediation and the nanotechnology industry.

## Introduction

Phenol, an essential organic solvent, is produced as a byproduct by many industrial processes, such as petroleum and oil refineries and pharmaceutical industries^1,2^. As an organic compound with toxic, mutagenic, and carcinogenic effects on organisms, it could pose a significant problem in water and groundwater due to its high solubility and toxicity^2,3^. In addition, it can be lethal when inhaled or ingested and harm cardiovascular and neurological systems^4,5^. Phenol is one of the most hazardous compounds and quite resistant to natural degradation, which is documented by the US Environmental Protection Agency (EPA) as a priority organic pollutant^4,6^.

While the World Health Organization (WHO) recommended that 1000 mg/L of phenol should be present in the drinking water, the amount of phenol in manufacturing wastes differs from 10 to 3900 mg/L, even reaching 6800 mg/L in severely contaminated effluent^7,8^. Consequently, effective treatment techniques must be used to lower the concentration of phenol to safe levels in refinery effluents. The remediation of phenol from wastewater can typically be accomplished by employing physicochemical or biological methods, which the latter believed to be the most effective and cost-effective methods^9^.

On the other hand, tellurium (Te) is a rare and toxic metalloid whose biological role has not been determined yet, which is found in trace constituents in the earth’s crust, with an abundance of only 1 to 5 mg/kg^10^. Long-term tellurium exposure can lead to several health issues, including dermatitis, weakness, headache, drowsiness, gastrointestinal symptoms, and respiratory irritation^11^. Te can be found in the environment in a variety of oxidation states, including Te^0^ (elemental Te), Te^2−^ (telluride), TeO_3_^2−^ (tellurite), and TeO_4_^2−^ (tellurate)^12,13^. Tellurite is believed to be one of the most harmful forms of Te oxyanions which can even harm microorganisms at a concentration of 1 mg/L. On the other hand, Te^0^ is less soluble and bioavailable, making it less toxic to microorganisms^14,15^.

Te has recently been employed in a variety of contemporary industrial domains, such as solar panels, textiles, metal alloys, glasses, electrical gadgets, new-generation rechargeable batteries, and cutting-edge materials, like Te-based quantum dots (QDs)^16,17^. Due to its scarcity and growing demand for industrialization, its price is expected to keep rising^18^. The European Union and the US Department of Energy have classified Te as a crucial element due to rising numbers of documented uses since its scarcity might jeopardize the advancement of innovative technology in energy and military fields^19^.

However, the presence of Te oxyanions in the disposed wastes and contaminated soils indicating the increased usage level has resulted in significant Te pollution. Furthermore, tellurite needs to be remediated because of the extensive usage of Te and its discharge into the environment^20^. Tellurite remediation using traditional methods is expensive and inefficient for large quantities of wastewater with compound organic debris and low metals volumes. Accordingly, microbial remediation is becoming more popular as a substitute for physicochemical techniques^13^.

A substantial source for additional Te recovery may come from the microbial remediation of tellurite into the less harmful Te nanoparticles (TeNPs), which offers a possible way for environmental Te bioremediation^21^. Their characteristics, including morphology, size, surface functionality, and crystallinity, are crucial in defining their prospective applications in various fields^22^.

The adverse impacts of tellurite and phenolic contaminants pose a serious problem due to their high toxicity to organisms. Therefore, the simultaneous bioremediation of tellurite and phenolic contaminants from industrial wastes such as petrochemical industries, which are often found together, is vital^15,23,24^.

The co-contaminant remediation of phenol and tellurite by microorganisms has thus far not been investigated. Microbial reduction of the tellurium oxyanions, in addition to the biodegradation of phenolic contaminants, can create the groundwork for energy and cost-efficient dual treatment procedure of aromatic compounds and metalloid oxyanions present in the industrial effluents.

Therefore, this study investigated the capability and efficiency of co-contaminant bioremediation of phenol and tellurite as two models of aromatic compounds and metalloid oxyanions. The isolated bacterium consumed phenol as its sole carbon source and energy, and tellurite was reduced to Te nanoparticles simultaneously. The biosynthesized TeNPs were extracted and characterized using dynamic light scattering (DLS), FE-SEM, energy-dispersive X-ray (EDX), X-ray crystallography (XRD), and Fourier-transform infrared spectroscopy (FT-IR).

## Material and methods

### Isolation and identification

EBL303 was isolated from disposed effluent in the Environmental Biotechnology Lab (EBL) of the University of Tehran. The capability of the strain in phenol degradation and tellurite reduction was evaluated qualitatively by culturing EBL303 in the Bushnell Haas (BH) medium^25^, consisting of 1000 mg/L NH_4_NO_3_, 1000 mg/L KH_2_PO_4_, 1000 mg/L K_2_HPO_4_, 20 mg/L CaCl_2_, and 200 mg/L MgSO_4_, 50 mg/L FeCl_3,_ supplemented with phenol (as a sole carbon source) and tellurite.

The 16S rRNA gene was amplified using extracted and purified total genomic DNA. PCR product was sequenced (Macrogen, South Korea), and the attained sequences were aligned employing BLAST with sequences existing in the GenBank database. The neighbor-joining method was used in order to build the phylogenetic tree by means of MEGA X^26^ software (bootstrap test of 1000 replicates). The obtained data were submitted to the NCBI databases under the accession number OM010344.

### Phenol and tellurite remediation in separate and co-contaminant cultures

All of the experiments were carried out in a 500 mL Erlenmeyer flask, including 100 mL (final volume) BH medium (pH = 7.0) in a shaker (180 rpm) at 30 °C for 98 h. BH was supplemented with 500 mg/L yeast extract (YE) in one set of experiments to evaluate the changes in the bacterium performance. 500 mg/L phenol as a sole carbon source and 0.5 mM potassium tellurite were added to without YE (YE-) and with YE (YE +) flasks of phenol and tellurite. Also, 5% (v/v) of the defined concentration of fresh inoculum was transferred to each flask. The efficiency of the bacterium was evaluated in six different culture conditions, including two phenol cultures (YE + and YE-), two tellurite cultures supplemented with 2500 mg/L glucose, and 500 mg/L yeast extract, and two mixed cultures of phenol and tellurite (YE + and YE-).

### Minimal inhibitory concentrations (MIC)

MIC was determined by inoculating 5% (v/v) of fresh cultures (OD_600_ ~ 0.1) into 5 mL of BH medium. 2500 mg/L of glucose was added to tellurite flasks as a carbon source. Tellurite and phenol were added to flasks at different concentrations of phenol (500–4000 mg/L) and tellurite (0.5–10 mM), and cells were incubated for 48 h with constant shaking (180 rpm) at 30 °C.

### Determining phenol and tellurite concentration

An adjusted rendition of a colorimetric assay represented by^27,28^ was utilized to assess the concentrations of phenol in which the reaction of phenolic compounds with 4-aminoantipyrine (20.08 mM) and potassium ferricyanide (8.34 mM) in sodium bicarbonate solution (0.25 M) produced a red dye. 1 mL of samples were centrifuged at 13,000 rpm for 2 min. Then, 900 μL of the supernatant (1:70 dilution) was added to 50 μL 4-aminoantipyrine and 50 μL potassium ferricyanide. The absorbance of the subsequent combination was assessed at 510 nm after 6 min.

According to a method described by^29^, NaBH_4_ (sodium borohydride) was used to evaluate the tellurite concentration in the media by a spectrophotometric method. The tellurite in the sample was reduced by freshly made NaBH_4_ solution (3.5 mM, final concentration), and its absorption was assessed at 500 nm. Phenol and tellurite concentrations were calculated through a standard curve.

### Kinetics assay of phenol and tellurite remediation

Considering the significance of predicting the contaminant level in the remediation procedures, kinetics studies were conducted to comprehend the phenol and tellurite remediation model by EBL303. In this assessment, three distinct experimental cultures containing BH medium were supplemented with 500 mg/L phenol, 0.5 mM tellurite accompanied by 2500 mg/L glucose as carbon source, the combination of phenol and tellurite, 500 mg/L yeast extract, and 5% (v/v) of fresh inoculum then incubated over 98 h. About 1 ml of the sample from all cultures, 1 mL from phenol-containing cultures, 2 mL from tellurite-containing cultures, and 3 mL from mix cultures were withdrawn at designated times (0, 5, 9, 24, 29, 33, 48, 56, 74, 98 h) to investigate biomass concentration (CFU/mL), phenol degradation, and tellurite reduction.

In order to achieve the described parameters, three mathematical models, including zero, first, and second-order, were investigated, and their time-series graphs were drawn. With the intention of determining the removal kinetics of phenol and tellurite, the following models were employed:

1 $$\left( {{\text{Zero}}\text{-}{\text{order}}} \right):\;{\text{Ct}} = {-}{\text{K}}_{0} {\text{t}} + {\text{C}}_{0} \;{\text{and}}\;{\text{T}}_{{{1}/{2}}} = {\text{C}}_{0} /{\text{2K}}_{0}$$

2 $$\left( {{\text{First}}\text{-}{\text{order}}} \right):\;{\text{ln Ct}} = {-}{\text{K}}_{{1}} {\text{t}} + {\text{lnC}}_{0} \;{\text{and}}\;{\text{T}}_{{{1}/{2}}} = {\text{Ln2}}/{\text{K}}_{{1}}$$

3 $$\left( {{\text{Second}}\text{-}{\text{order}}} \right):\;{1}/{\text{Ct}} = {\text{K}}_{{2}} {\text{t}} + {1}/{\text{C}}_{0} \;{\text{and}}\;{\text{T}}_{{{1}/{2}}} = {1}/{\text{C}}_{0} {\text{K}}_{{2}}$$

Which t stands for time, Ct for concentration at time t, C0 for initial concentration, and K for removal rate constant^30,31^.

### Preparation of TeNPs

The EBL303 was employed to synthesize TeNPs. The TeNPs were extracted from the bacterial cells following the protocol described by^32^ with some modifications. In a typical experiment, 100 mL of BH medium was prepared and supplemented with 0.5 mM tellurite, 500 mg/L phenol, 500 mg/L yeast extract, and 5% (v/v) fresh inoculums (180 rpm at 30 °C). By centrifuging the bacterial biomass at 6000 × g for 10 min after 48 h of incubation, the TeNPs-containing bacteria were separated from the medium. The separated biomass was washed twice by centrifugation in deionized water. Then, they were frozen and crushed by adding liquid nitrogen. After that, liquid nitrogen was employed to freeze and crush the biomass. The crushed bacteria were collected and washed by centrifugation at 10,000 × g for 5 min three times with 1.5 M Tris/HCl buffer (4 °C, pH 8.3) with 1% SDS and one more time with deionized water. Deionized water was added to the resulting slurry containing TeNPs, and the crushed cell remains. In order to prevent aggregation, the suspended TeNPs were treated with 5 min of 100 W ultrasonication in cold water. 12 mL of the resulted slurry was combined with 6 ml of 1-Octanol and was shaken robustly. After centrifugation at 2000 × g (5 min), it was kept at 4 °C for one day. Then, the mid-layer (cell debris) and upper phase (1-Octanol) were discarded prudently, and deposited NPs were washed with deionized water. 50 mM of Tris–HCl (4 °C, pH 7.4) was added to the deposited NPs, and purified NPs were stored at 4 °C^32^. Refined TeNPs were used for characterization analyses.

### Characterization of TeNPs and cell morphology

In order to confirm and characterize the biosynthesized TeNPs and their structures, transmission electron microscopy (TEM), dynamic light scattering (DLS), field emission scanning electron microscopy (FE-SEM), energy-dispersive X-ray (EDX), Fourier-transform infrared spectroscopy (FT-IR), and X-ray crystallography (XRD) analyses were carried out. The shape and size of TeNPs inside the cells were visualized utilizing TEM (Philips EM208S) at 100 kV after the cells were fixed, dried, and thin sections were taken after embedding the sample in LR white resin. The particle size distribution of the TeNPs was determined by the DLS technique (Zetasizer Ver. 6.01, Malvern Instruments Ltd). Employing an X-ray diffractometer (Philips PW1730), the crystallinity of TeNPs and structure were studied. The functional groups capped to the TeNPs were examined using FT-IR (Thermo, AVATAR) analysis. The cell morphology was observed using SEM (VEGA3 TESCAN) at 20 kV after the sample was fixed, dried, and coated with gold. To observe the size and surface features, NPs were dried on aluminum foil and then coated with gold. Samples were analyzed using an FE-SEM (ZEISS Sigma 300) operated at 10 kV and equipped with an EDX. EDX was performed on the same samples to determine the elemental composition of NPs.

### Statistical analysis

Statistical analysis of the experimental outcomes was performed in R Studio (version 3) utilizing R version 4.1.2. All the bioremediation experiments were performed with three replicates. The normality of the distribution and homogeneity of variance of the analyzed data were evaluated applying the Kolmogorov–Smirnov and Levene’s test, which showed that the data had a normal distribution with homogenous variances. One-way ANOVA and Tukey’s test was implemented to compare the differences between the cultures.

## Result and discussion

### Isolation and identification of EBL303

**Figure 1:**
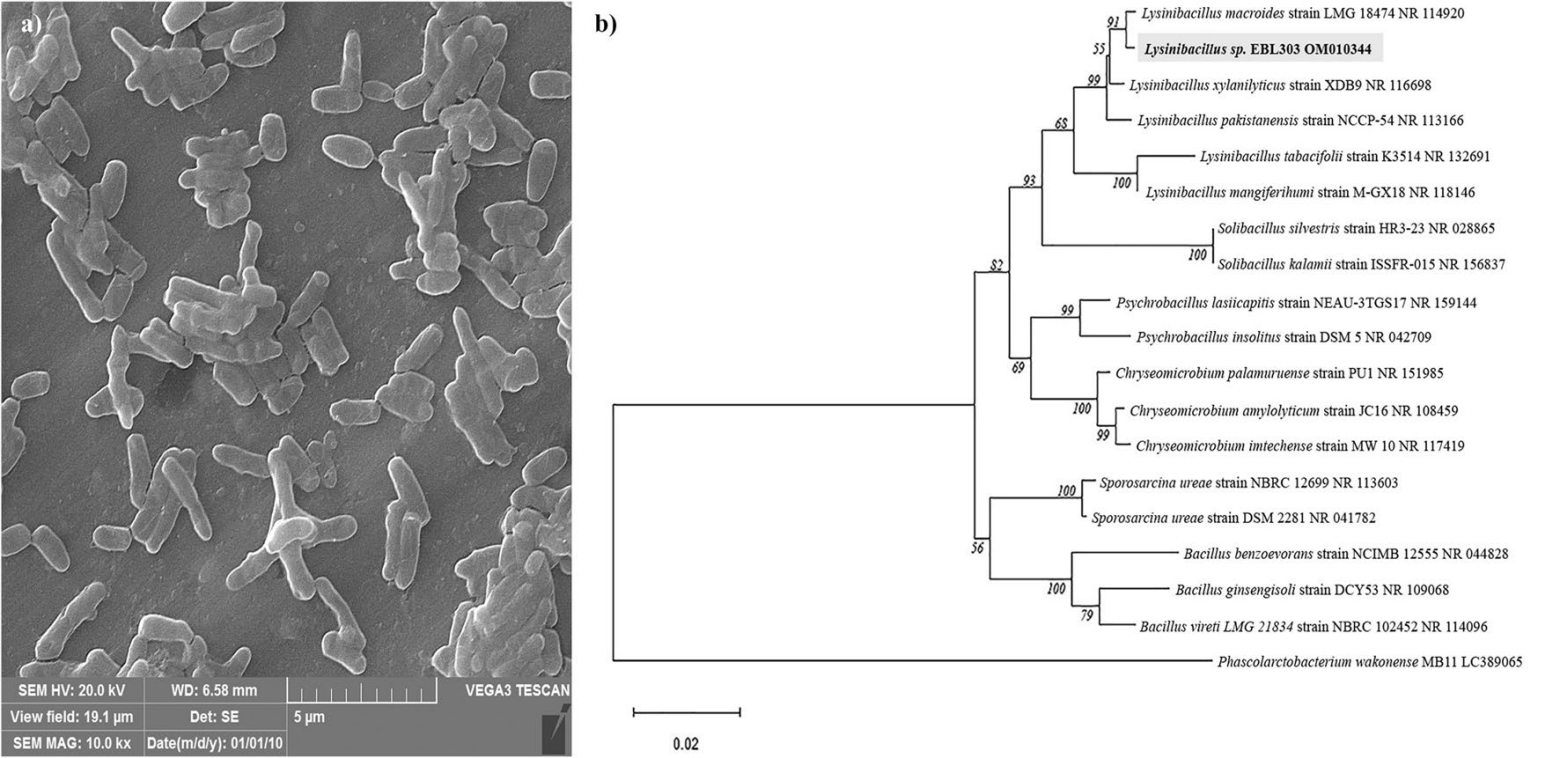
SEM micrograph of strain EBL303 (**a**) and Phylogenetic tree of *Lysinibacillus* sp. EBL303 (**b**) and its related sequences from the NCBI database.

EBL303 was a bacterial strain isolated from EBL, and its potential for simultaneous remediation of phenol and tellurite was investigated in BH with different concentrations of phenol as a sole carbon source and tellurite. EBL303 was able to degrade phenol, and its black color indicated tellurite reduction and formation of TeNPs. The EBL303 was identified morphologically as a gram-positive bacterium with rod-shaped and pinkish punctiform colonies on the medium. The SEM investigation revealed that the EBL303 cells were short rods with a size of approximately 2.5 μm × 0.5 μm (Fig. 1a).

Analyzing the growth capability in the presence of various NaCl concentrations showed that EBL303 grew in up to 5% NaCl. In these circumstances, this halotolerant capability of the bacterium to grow in a wide variety of salt concentrations makes it a worthy candidate for biotransformation and bioremediation of toxic materials and metalloids from industrial wastewater^33^.

After determining and comparing the EBL303 16S rRNA gene sequence with other strains in the NCBI database, a phylogenetic tree was constructed (Fig. 1b). The findings indicated that strain EBL303 is a member of the *Lysinibacillus* genus, which shares the most sequence similarities with *Lysinibacillus macroides* (99.30%). Therefore, the isolated strain was designated as *Lysinibacillus* sp*.* EBL303. As far as we are aware, only one research has reported the *Lysinibacillus* genus’s ability in tellurite reduction^34^, despite the fact that the phenol degradation ability of this genus is well known^35–37^.

### Phenol and tellurite MICs of *Lysinibacillus* sp. EBL303

The growth of *Lysinibacillus* sp*.* EBL303 was suppressed at tellurite and phenol concentrations of 7 mM and 2500 mg/L, respectively. The phenol and tellurite tolerance of this strain was relatively high compared to other studies. According to^38^, *Bacillus cereus* MTCC9817 growth was completely inhibited at a 2000 mg/L or higher phenol concentration. In another study, 32 yeast strains were evaluated, and none could grow in phenol concentrations higher than 600 mg/L^39^. In some similar studies, MICs of 5 mM in *Lysinibacillus* sp.^34^, 6 mM in *Pseudomonas pseudoalcaligenes*^40^, 12 mM in *Salinicoccus* sp.^41^, and 13 mM in *Escherichia* sp.^18^ for tellurite were reported.

### Phenol and tellurite bioremediation and their proposed mechanism

Remediation assays of 500 mg/L phenol and 0.5 mM tellurite were performed in separate cultures in order to assess the bioremoval capability of *Lysinibacillus* sp*.* EBL303. Furthermore, an experiment was performed on a culture supplemented with a combination of 500 mg/L phenol and 0.5 mM tellurite in order to assess the impact of aromatic compounds and metalloid oxyanions co-contamination on the remediation efficiency of *Lysinibacillus* sp. EBL303. Additionally, the effect of using yeast extract as a supplement was studied on phenol and tellurite remediation.

Table 1 displays the findings from the assessment of the remediation of phenol, tellurite, and phenol/tellurite combination. It is worth mentioning that we did not observe any phenol and tellurite remediation in the absence of bacteria, indicating the remediation can only be attributed to the bacterium’s activity. As shown in Table 1, YE + s displayed the highest phenol and tellurite remediation efficiencies in separate cultures, with 99.92 and 100% within 74 and 56 h, respectively. It should be noted that the lower extent of tellurite reduction in YE- culture compared with YE + culture could be related to insufficient carbon source in this batch (only 500 mg/L yeast extract).

**Table 1:** The yield of phenol and tellurite removal in different cultures in 98 h.

	YE-		YE +	
	Phenol degradation (%)	Tellurite reduction (%)	Phenol degradation (%)	Tellurite reduction (%)
Separate cultures	99.24 ± 0.46 a	73.33 ± 8.14 a	99.92 ± 0.13 a	100 ± 0.0 c
Co-contaminant cultures	99.04 ± 0.71 a	37.07 ± 6.29 b	99.92 ± 0.13 a	73 ± 3.67 a

Values are mean (n = 3) ± SD. Different alphabets in phenol and tellurite columns represent significance at *p* < 0.05 after applying post hoc Tukey’s test.

In similar studies, *Lysinibacillus* sp. reduced 70% of 0.5 mM of tellurite in 48 h^34^, and *Raoultella* sp. reduced 100% of 0.5 mM of tellurite in 30 h^18^. In another study, a bacterial strain belonging to *Shinella* sp. reduced almost 100% of tellurite in 0.3 and 0.5 mM concentrations within 28 and 44 h, respectively^15^. Some bacteria, like *Acinetobacter* sp. and *Pseudomonas* sp., were able to degrade all of the 500 and 700 mg/l of phenol within 18 and 30 h, respectively^42,43^. According to^23^ findings, *Rhodococcus* sp. degraded 401, 1025, and 1806 mg/L of phenol entirely within 14, 20, and 38 h. In another study, a co-culture of *Stenotrophomonas* sp. and *Advenella* sp. were able to completely degrade 1200 mg/L of phenol in 70 h^44^. Also, Essam et al.^45^ reported that *Alcaligenes* sp. could completely degrade 1200 mg/L phenol in 40 h.

Besides, in co-contaminant bioremediation, YE + cultures performed better in removing phenol and tellurite within 98 h. This might be due to the fact that yeast extract has a significant amount of riboflavin, an electron shuttle that can be used to accelerate bioremediation^46^. Also, compared to the YE + cultures, black TeNPs were less prevalent in the YE- cultures. In a study by^46^, the effect of shuttles on selenite bioreduction was investigated in *Shewanella oneidensis* MR-1. The results showed that the pathway of selenite bioreduction was changed in this bacterium in the presence of riboflavin, and selenite nanoparticle was produced extracellularly. Since selenite and tellurite have many common characteristics, the presence of some TeNPs in the culture medium can be related to this matter.

Interestingly, when both phenol and tellurite were present, all co-contaminant cultures reduced tellurite at a significantly lower rate (P < 0.05) within 98 h. Meanwhile, the amount of phenol degradation by co-contaminant cultures was almost the same (99.04 and 99.92% from 99.24 and 99.92%, respectively) in 98 h. In a similar study, a co-culture of *Phanerochaete* sp. and *Delftia* sp. reduced about 10 mg/L of selenite in 72 h with simultaneous degradation of 100% of 400 mg/L phenol. Overall, it can be suggested that the bioremediation of aromatic compounds and metalloid oxyanions is more successful in the separate and YE + cultures than in the co-contaminant and YE- cultures within 98 h. Even though phenol was degraded entirely in all cultures, the removal rate was faster in the separate and YE + cultures.

**Figure 2:**
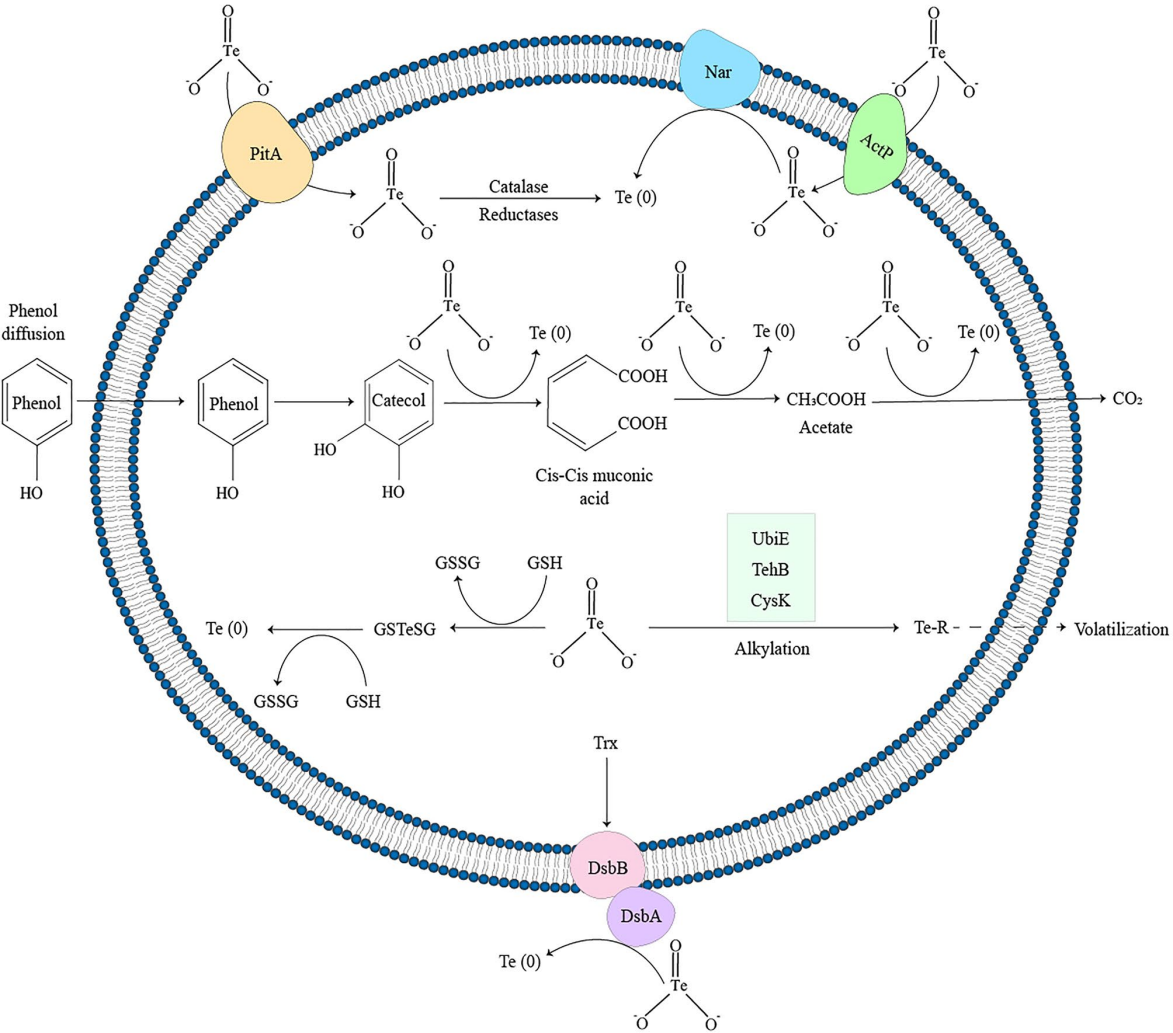
The proposed mechanism for degradation of phenol coupled with reduction of tellurite to TeNPs by *Lysinibacillus* sp. EBL303 (created by Adobe Illustrator 2022, https://www.adobe.com/products/illustrator.html).

The proposed mechanism for simultaneous bioremediation of phenol and tellurite is shown in Fig. 2. There are various transporters responsible for entering the oxyanions into the bacteria, among which two transporters, acetate permease (ActP)^47^ and phosphate transporter (PitA)^48^, are believed to be involved in the uptake of tellurite by the bacteria.

Various mechanisms such as bioreduction by reductases, including transmembrane and intracellular reductases^49^ as well as the thioredoxin (Trx) and glutathione (GSH) system^50,51^ can be responsible for transforming tellurite into a less toxic compound. As the only carbon source, phenol can easily be dissolved in the membrane and enters the cell. Inside the cell, biodegradation begins with phenol being converted into catechol by phenol hydroxylase. Catechol is further degraded to cis,cis-muconate via catechol 1,2-dioxygenase^52^. The resulting compound can be metabolized to Krebs cycle intermediates through the beta-ketoadipate pathway. Due to these reactions, some electrons and various intermediates are produced^52^. The obtained electrons can eventually lead to the reduction of tellurite and TeNP biosynthesis. In addition, the alkylation of tellurite and the production of its volatile compounds^53^ are other reactions that can result in the removal of tellurite by bacteria.

### The removal kinetics and growth rate of *Lysinibacillus* sp. EBL303

**Figure 3:**
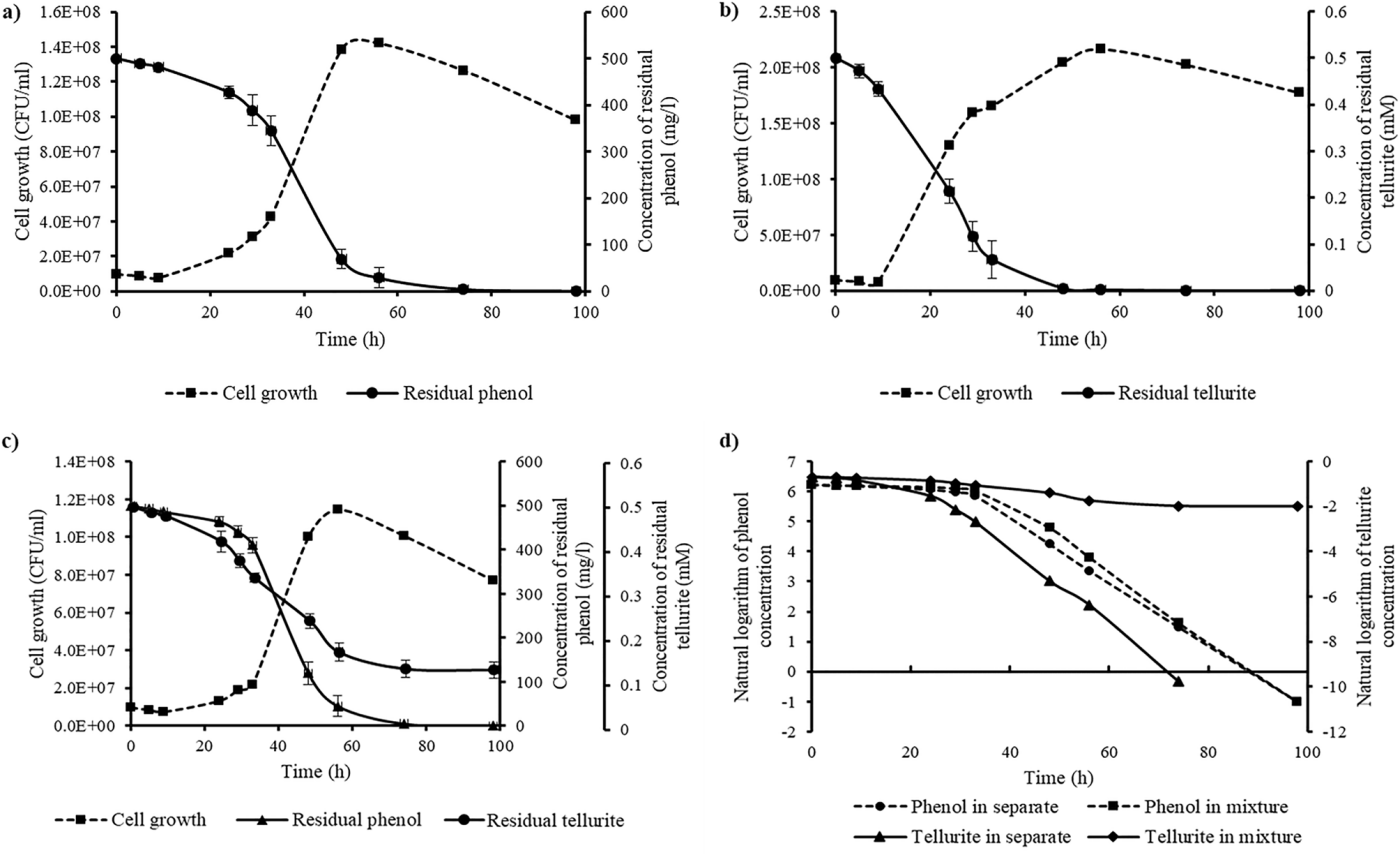
(**a**–**c**) the growth rate and remediation yield of *Lysinibacillus* sp*.* EBL303, respectively, in phenol (500 mg/L), tellurite (0.5 mM), and phenol (500 mg/l)/tellurite (0.5 mM) mixture; (**d**) the first-order removal model of phenol and tellurite in separate and mixture. Values are mean ± SD of three replicates.

In bioremediation, the removal kinetics of contaminants is recognized as an imperative element that is a valuable method for monitoring, predicting, and simplifying biological processes^30^. The determined removal kinetics of phenol and tellurite and their growth curves are presented in Fig. 3. From this graph, a direct connection can be seen between the growth and remediation of phenol and tellurite. Additionally, the reliance on remediation with growth rate is consistent with the findings of^18,23^. Degradation of phenol and tellurite reduction displayed the same pattern, which indicates that phenol and tellurite were remediated simultaneously (Fig. 3c).

Three main growth periods, including lag, exponential, and death phases, were detected during the remediation of phenol in separate cultures (Fig. 3a). During the lag phase, the bacterial strain did not significantly degrade phenol. The maximum rate of phenol degradation was observed in the exponential phase. And in the death phase, the number of living bacteria decreased, and phenol was utterly degraded. Similar results were obtained by^23^. In the remediation of tellurite in separate cultures, the same number of aforementioned growth main phases were detected (Fig. 3b). By the end of the lag phase, tellurite had been significantly reduced to TeNPs by the strain. During the exponential phase, tellurite was entirely reduced by the microorganism. And a death phase after consuming all of the carbon sources and a complete reduction of tellurite. A similar pattern of cell growth and tellurite reduction was reported by^18^.

Figure 3d displays the first-order model of the phenol and tellurite remediation. The removal rate constant (K), half-life period (T_1/2_), and correlation coefficient (R^2^) of all employed removal kinetics models in both cultures are presented in Tables 2 and 3. The first-order removal model displayed a higher correlation coefficient in both cultures, indicating that the first-order kinetics model fitted well with phenol and tellurite remediation. Table 3 shows that the removal rate constant of phenol degradation was reduced to 0.0732 per h, and the half-life increased to 9.469 h, which shows phenol degradation rate is slower in co-contaminant cultures. Furthermore, in the co-contamination cultures, the removal rate constant of tellurite was reduced to 0.0161 per h, and the half-life increased to 43.05 h. In other words, it is reasonable to infer that in cultures containing both phenol and tellurite, a portion of bacterial energy is aimed to lower the phenol toxicity, which results in the reduction of growth.

**Table 2:** Kinetic parameters for 500 mg/L phenol and 0.5 mM tellurite removal in separate cultures.

Parameters	Phenol	Tellurite
Zero-order equation	Ct = − 6.4279 t + 515.14	Ct = − 0.0078 t + 0.4423
K_0_ (per h)	6.4279	0.0078
T_1/2_ (h)	40.07	28.35256
R^2^	0.861	0.8354
First-order equation	LnCt = − 0.0749 t + 7.2679	LnCt = − 0.1222 t + 0.429
K_1_ (per h)	0.0749	0.1222
T_1/2_ (h)	9.254301	5.672236
R^2^	0.9049	0.9289
Second-order equation	1/Ct = 0.0193 t − 0.4236	1/Ct = 156.23 t − 2791.3
K_2_ (per h)	0.0193	156.23
T_1/2_ (h)	0.045562	0.05597
R^2^	0.5085	0.4484

**Table 3:** Kinetic parameters for 500 mg/L phenol and 0.5 mM tellurite removal in co-contamination cultures.

Parameters	Phenol	Tellurite
Zero-order equation	Ct = − 6.506 t + 541.15	Ct = − 0.0045 t + 0.4992
K_0_ (per h)	6.506	0.0045
T_1/2_ (h)	41.58853	55.46667
R^2^	0.855	0.9215
First-order equation	LnCt = − 0.0732 t + 7.3572	LnCt = − 0.0161 t − 0.6121
K_1_ (per h)	0.0732	0.0161
T_1/2_ (h)	9.469224	43.05262
R^2^	0.8721	0.9332
Second-order equation	1/Ct = 0.0191 t − 0.4229	1/Ct = 0.0657 t + 1.3816
K_2_ (per h)	0.0191	0.0657
T_1/2_ (h)	0.045164	21.02892
R^2^	0.4996	0.9154

Tellurite slightly hindered the metabolism and bacteria’s growth and the degradation of phenol in the co-contaminant cultures; however, the difference was not significant. Only after 24 h, TeNPs begin to appear in the co-contaminant cultures, demonstrating that the bacteria used phenol degradation intermediates to reduce tellurite to TeNPs. However, the reduction was observed from the beginning in the absence of phenol. On the other hand, growth is a result of phenol degradation, and the best rate of phenol degradation was observed during the exponential growth phase, indicating that phenol degradation improves as the growth rate rises. The maximum growth in co-contaminant cultures is 1.15E + 08 CFU/ml, which is 1.9 times lower than growth in separate tellurite cultures. In other words, the tellurite reduction decreases as the growth declines since fewer enzymes necessary for the process are produced. Although maximum cell growth between tellurite in separate and mixed cultures showed a significant difference, no significant difference was identified in phenol cultures, despite the fact that the cell growth in co-contaminant culture lagged slightly behind the separate phenol culture, probably due to the toxicity of tellurite to the bacterium.

Phenol and tellurite batches showed different lag phases, which for phenol batches attained within 24 h while for tellurite were within about 12 h of incubation. The delay in the growth and remediation can be credited to the bacterial cells’ adaptability to phenol and tellurite and the bioconversion of phenol to intermediate chemicals, which are relatively more accessible and less toxic. The lag phase extended from 12 to 33 h in co-contaminant cultures, as shown in Fig. 3c, which implies the presence of another toxic compound, therefore, a decrease in tellurite reduction.

Since this is the first study investigating the kinetics and co-contaminant bioremediation of phenol and tellurite, more experiments with various cultures, concentrations, and combinations are required to evaluate and optimize the simultaneous bioremediation of aromatic compounds and metalloid oxyanions.

### Characterization of produced tellurium nanoparticles

**Figure 4:**
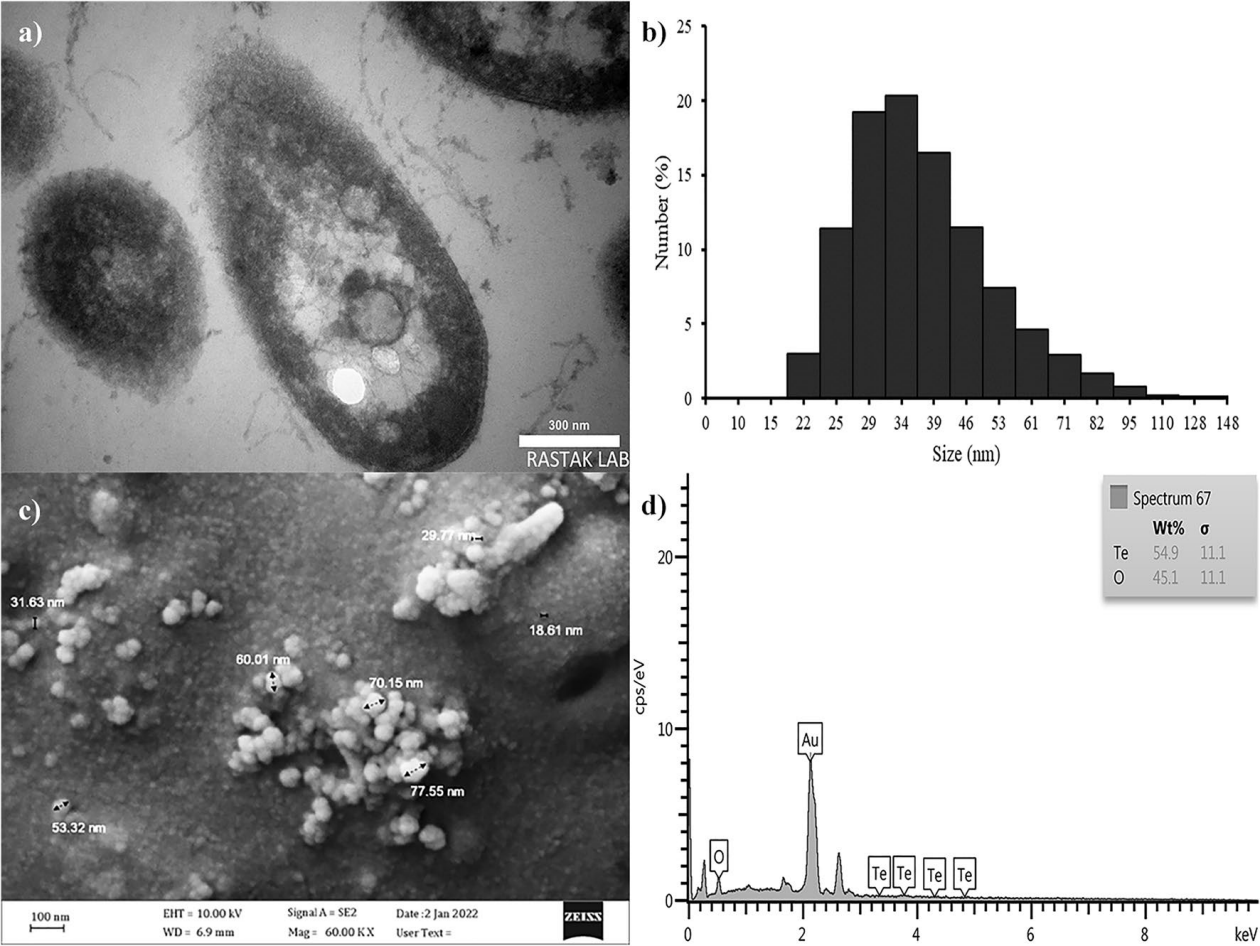
TEM micrograph (**a**), DLS analysis (**b**), FE-SEM image (**c**), and EDX spectrum (**d**) of the extracted TeNPs synthesized by *Lysinibacillus* sp. EBL303.

The Amorphous TeNPs were intracellularly synthesized by bacterial strain, which was confirmed by TEM images (Fig. 4a) and comparing SEM images of the cell extracts containing TeNPs with intact bacterial SEM images (Supplementary Fig. S1). The TEM micrograph revealed that these nanospheres were often associated with the cell membrane (Fig. 4a), similar to findings reported by^54,55^, which indicates that Te nanospheres were formed via reduction mechanisms occurring inside the cell. Additionally, the formation of these nanoparticles(black points inside different parts of the cell) in different parts of cells indicates that several nucleation points could exist within the cytoplasm for initiating tellurite reduction.

The DLS analysis showed that the extracted TeNPs varied in size from 22 to 148 nm, with 34 nm NPs having the highest frequency (Fig. 4b). Based on DLS results, the obtained TeNPs of this study were smaller than those generated by *Aureobasidium pullulans*^56^, *Aspergillus welwitschiae*^57^ and *Ochrobactrum* sp.^58^. The FESEM images of TeNPs showed that the spherical nanoparticles tended to agglomerate and created aggregates of various sizes formed of smaller particles due to their high surface energy and lack of capping agents^13,59^. On the other hand, single nanodots could be seen around the aggregates, which were much smaller than the aggregates and the result of DLS analysis (Fig. 4c). This phenomenon is also reported by^56^, which confirms the fact that when NPs tend to accumulate, DLS is not an accurate test for measuring the size of NPs. The composition of the black nanoparticles was verified to be elemental Te based on EDS data (Fig. 4d) which was similar to the findings of^60^.

**Figure 5:**
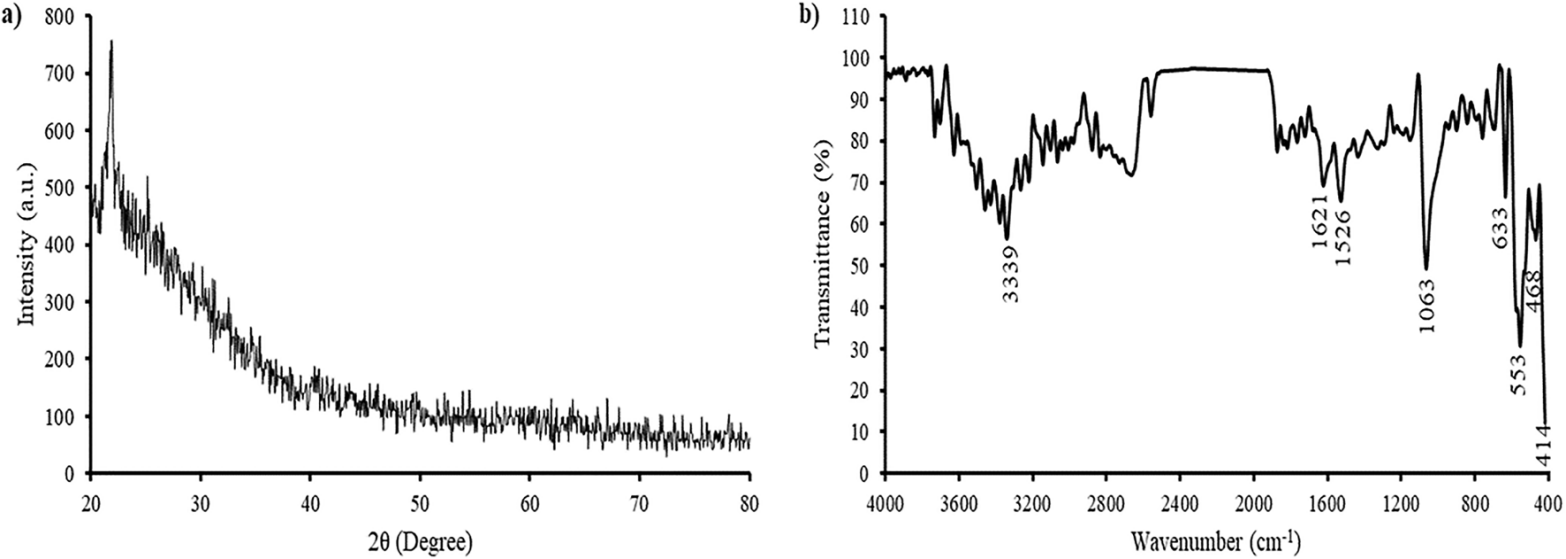
XRD pattern (**a**) and FTIR analyses (**b**) of biosynthesized TeNPs.

The XRD spectrum of the TeNPs revealed broad peaks devoid of any sharp Bragg reflections (Fig. 5a), indicating a non-crystalline nature of the TeNPs. A similar result was reported by^61^, which stated that the XRD spectrum of selenite NPs showed no distinct peak. Figure 5b shows a FTIR spectrum of biosynthesized TeNPs with absorption bands at 3339 cm^−1^, which can be attributed to a hydroxyl group^62^, 1621 cm^−1^ to amide group^63^, 1526 cm^−1^ to an aromatic ring^62^, 1063 cm^−1^ suggests primary amine (CN stretch)^63^, 633 cm^−1^ can be related to disulfide compounds^62^, and 553 cm^−1^ can correspond to aliphatic compound^63^, which indicates the surface of the NPs were capped by proteins and lipids^61^.

## Conclusion

For the first time, this study reports the co-contaminant bioremediation of phenol (as an aromatic compound) and tellurite (as a metalloid oxyanion), along with biosynthesis and subsequent characterization of TeNPs produced by *Lysinibacillus* sp*.* EBL303. Which displays the feasibility of pure bacterial culture for bioremediation of polluted sites and TeNPs biosynthesis. Both phenol and tellurite exhibited inhibitory behavior, and their removal kinetics could be correlated well by the first-order modeling of removal kinetics. The results of separate cultures have shown that the initial phenol and tellurite concentrations of 500 mg/L and 0.5 mM could be entirely removed in 74 and 56 h, respectively. In co-contamination cultures, *Lysinibacillus* sp*.* EBL303 remediated 99.92 and 73% of phenol and tellurite, respectively, in 98 h, which is a promising result for the simultaneous remediation of two toxic compounds. Separate and YE + cultures showed higher efficiency in phenol and tellurite remediation than co-contaminant and YE- within 98 h. FE-SEM, EDX, DLS, FTIR, and XRD results confirmed that the purified TeNPs were covered with proteins and lipids with a nanoscale non-crystalline structure. This method offers an inexpensive and green system for the simultaneous remediation of phenol and tellurite in contaminated sites and TeNPs synthesizing for the nanotechnology industry. Although more research is needed to enhance the bioremediation efficiency and biosynthesis rate of TeNPs, these findings can be utilized to develop a practical bioremediation process, such as using bioreactors to treat wastewaters contaminated with aromatic and metalloid compounds.

## Supplementary Information

Supplementary Information.

## Data Availability

The datasets generated and/or analyzed during the current study are available in the NCBI repository. Accession number: OM010344 and link to seq in NCBI site: https://www.ncbi.nlm.nih.gov/nuccore/OM010344.
