# Supplementary material for: Simultaneous bioremediation of phenol and tellurite by Lysinibacillus sp. EBL303 and characterization of biosynthesized Te nanoparticles
Source: Sci Rep. 2023 Jan 23;13:1243. doi: 10.1038/s41598-023-28468-5 (PMC9870877; doi:10.1038/s41598-023-28468-5)
Supplement: Supplementary file 1 — Supplementary Information. [file 41598_2023_28468_MOESM1_ESM.pdf]

Simultaneous bioremediation of phenol and tellurite by *Lysinibacillus* sp. EBL303 and characterization of biosynthesized Te nanoparticles

Running Head: Remediation of model aromatic compounds and metalloid oxyanions

Firooz Hosseini[1], Elham Lashani[1], Hamid Moghimi[1*]

1- Department of Microbial Biotechnology, School of Biology, College of Science, University of Tehran, Tehran, Iran

Corresponding author: Hamid Moghimi

Department of Microbial Biotechnology, School of Biology, College of Science, University of Tehran, Tehran, Iran.

Tel: +98-21-66113314

Fax: +98-21-66415495

Postal code: 1417864411

Email: hmoghimi@ut.ac.ir

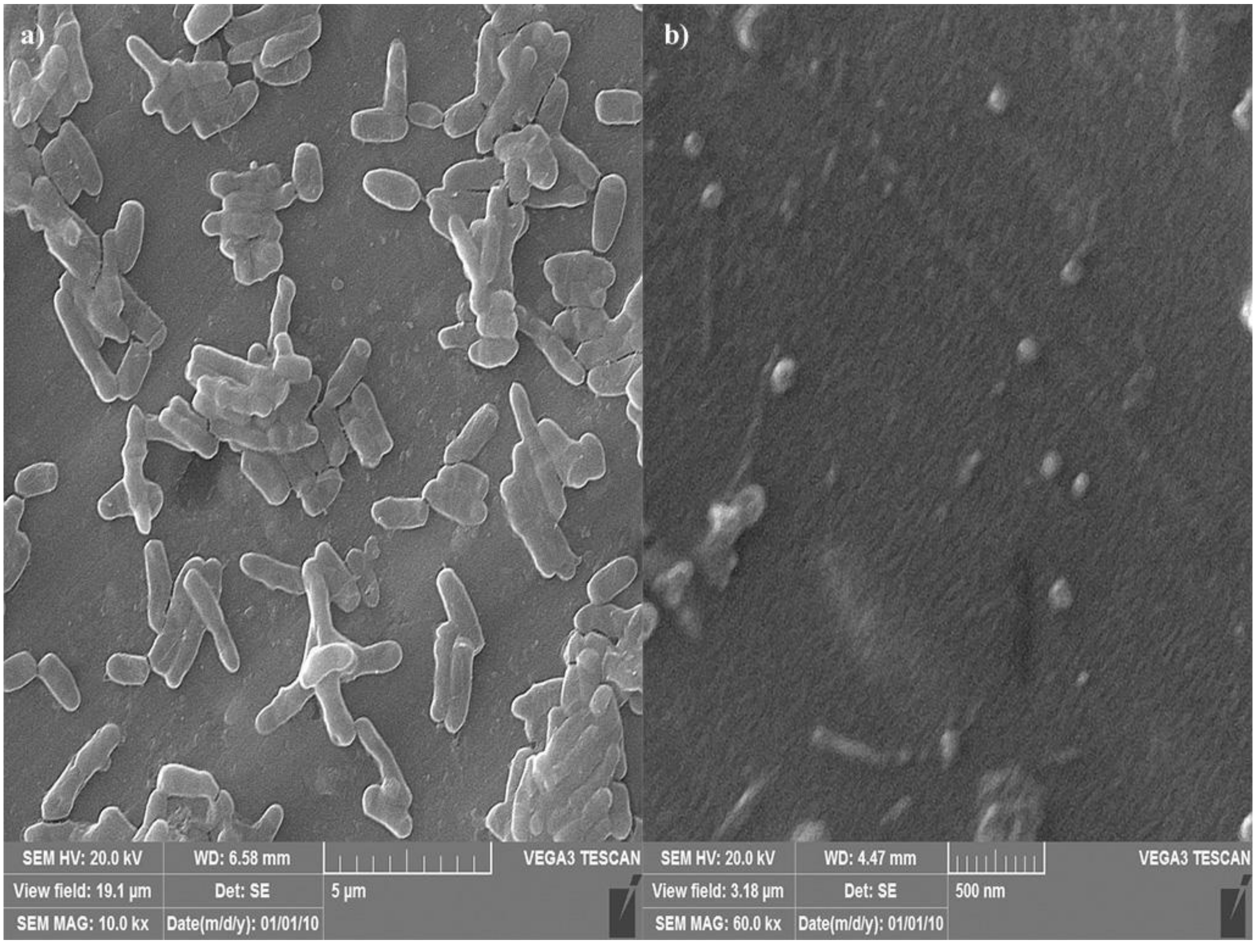

Supplementary Figure S1. SEM micrographs of strain EBL303 before (a) and after cell disruption (b), which showed that TeNPs were synthesized intracellularly.
